# Social learning solves the problem of narrow-peaked search landscapes: experimental evidence in humans

**DOI:** 10.1098/rsos.160215

**Published:** 2016-09-07

**Authors:** Alberto Acerbi, Claudio Tennie, Alex Mesoudi

**Affiliations:** 1 School of Innovation Sciences, Eindhoven University of Technology, Eindhoven, The Netherlands; 2 School of Psychology, University of Birmingham, Birmingham, UK; 3 Department of Biosciences and Human Biological and Cultural Evolution Group, University of Exeter, Penryn, UK

**Keywords:** social learning, cultural evolution, cultural transmission

## Abstract

The extensive use of social learning is considered a major reason for the ecological success of humans. Theoretical considerations, models and experiments have explored the evolutionary basis of social learning, showing the conditions under which learning from others is more adaptive than individual learning. Here we present an extension of a previous experimental set-up, in which individuals go on simulated ‘hunts’ and their success depends on the features of a ‘virtual arrowhead’ they design. Individuals can modify their arrowhead either by individual trial and error or by copying others. We study how, in a multimodal adaptive landscape, the smoothness of the peaks influences learning. We compare narrow peaks, in which solutions close to optima do not provide useful feedback to individuals, to wide peaks, where smooth landscapes allow an effective hill-climbing individual learning strategy. We show that individual learning is more difficult in narrow-peaked landscapes, but that social learners perform almost equally well in both narrow- and wide-peaked search spaces. There was a weak trend for more copying in the narrow than wide condition, although as in previous experiments social information was generally underutilized. Our results highlight the importance of tasks’ design space when studying the adaptiveness of high-fidelity social learning.

## 1. Introduction

Social learning—defined as changes in behaviour due to the observation of, or interaction with, another individual or their products [1,2]—is the focus of much recent work in the human evolutionary sciences. The extensive utilization of social learning in humans has been claimed to be one—or, perhaps, the main—reason for the enormous ecological success of our species [3–5]. Compared with other species, it is argued, humans possess a particularly faithful copying system that allows the preservation and accumulation of complex technological and cultural traits [6,7], as a rich set of ‘social learning strategies’ [8,9] or ‘transmission biases’ [10,11] that contribute to the adaptiveness of social learning.

These claims are supported by extensive theoretical modelling that has explored the evolutionary basis of social learning, identifying the expected conditions under which social learning is adaptive relative to individual learning and genetic adaptation [10,12–15], and the adaptiveness of specific transmission biases such as copying successful or prestigious demonstrators (success or prestige bias) [10] or majority behaviours (conformist bias) [16,17].

The assumptions and predictions of these models have also begun to be tested experimentally in the laboratory with real people. These experiments broadly confirm theoretical expectations, such as that people copy others when environments are relatively stable such that others’ solutions are not out of date [18,19], and employ success-biased [20,21] and conformist [17,22] social learning when appropriate. Nevertheless, puzzling anomalies also arise from experiments, such as that many people copy less than is optimal [18,21,23–25].

One limitation of many of these models and experiments is the simplicity of the behavioural ‘design space’ that determines pay-offs. This often constitutes a choice between one of two discrete options, one of which gives higher pay-offs on average than the other [12–15,18,20]. Yet much real-life social learning, particularly in complex domains such as human technology [26,27], is likely to occur when there are multiple potential solutions to problems, where those solutions have varying maximum pay-offs, and even partial solutions can provide some information about how close one is to a solution. In this sense, the ‘design space’ looks more like an adaptive landscape [28] with one or more locally optimal peaks of varying maximum cultural ‘fitness’.

**Figure 1.**
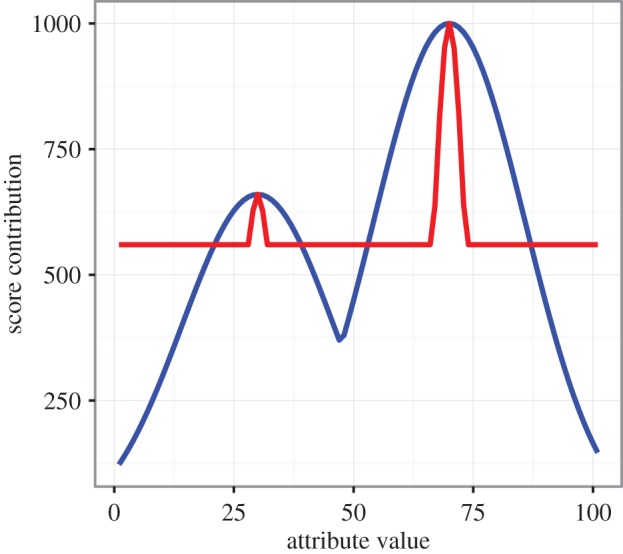
Examples of a wide- (blue line) and a narrow-peaked (red line) search landscape. The two peaks correspond to two arbitrary values (30 and 70) of the attribute. The search landscape shown here was used for the continuous attributes of the arrowhead, i.e. height, width and thickness, but with different optimal values for each. The red line has an enforced minimum of 560 calories to ensure that there is equal area under both lines.

In a series of laboratory experiments, Mesoudi and co-workers [21,29–31] have explored how people learn within such a multimodal adaptive landscape, using a task designed to simulate real-life human technological evolution. Here, participants design a ‘virtual arrowhead’ via a computer program. On each of a series of ‘hunts’, they can improve their arrowhead either by directly manipulating the arrowhead’s attributes (height, width, thickness, shape and colour), i.e. via individual learning, or by copying the arrowhead attributes of another participant, i.e. via social learning. On each hunt, participants receive a score in calories, representing their hunting score, based on their arrowhead design. Three of the attributes—height, width and thickness—are continuous and are each associated with bimodal fitness functions (e.g. figure 1, blue line). The overall hunt score is the weighted sum of the three fitness functions (plus the fitness function from the discrete shape attribute, which is unimodal; colour, the remaining attribute, is neutral and does not affect fitness). This generates a multimodal adaptive landscape with multiple (2^3^=8) locally optimal peaks of varying maximum pay-offs. The highest peak, located at the higher peak (e.g. 70 in figure 1) for all three attributes, gives a maximum hunt score of 1000 calories (plus or minus some small amount of random feedback error).

A key finding of these studies is that success-biased social learning (i.e. copying the design of a high-scoring other) in combination with individual learning is more adaptive than individual learning alone [29,30]. This is because pure individual learners get trapped on locally optimal but globally suboptimal peaks. Success-biased social learning allows individuals to ‘jump’ to higher-fitness peaks found by other, more-successful participants. This holds when social learning occurs after a period of enforced individual learning [29,30], when both individual and social learning is possible throughout the experiment [30], and when participants can copy from a separate group of individual-learning-only demonstrators [21,31] (although in each case, as noted above, not all participants copy others as much as they should do if they were maximizing pay-offs). The advantage of social learning is increased when an exogenous cost is imposed on individual learning [29], which acts to inhibit exploration of the adaptive landscape. The advantage is eliminated when the environment is unimodal [30], because pure individual learners can now easily find the single optimal peak using a simple hill-climbing (win-stay-lose-shift) algorithm [32].

The last observation depends on the fact that a hill-climbing strategy is effective for ‘smooth’ peaks, where individuals receive constant and reliable feedback on whether their changes brought them closer or not to the optimal solution. However, in many situations, and probably in the majority of modern technological tasks, this feedback is weak or non-existent. An example is tying a Windsor knot: correctly performing, say, 9 actions out of the necessary 10 does not produce a 90% correct Windsor knot, but is likely to produce an unusable object which does not tell the knot-learners how close they are to the right solution [33]. In sum, one factor that is missing from these experimental studies is a consideration of how the width of the fitness peaks affects social learning. In a previous model [34,35], Acerbi, Tennie and co-workers found that social learning is particularly useful in narrow-peaked landscapes, i.e. for problems in which solutions that are close to the optimum do not provide reliable feedback about how close one is to the peak. In wide-peaked landscapes, by contrast, while social learning can speed up the process of finding the correct solution, individual learning is also effective, as behavioural modifications provide reliable feedback to learners. A similar prediction can be derived from previous experimental work linking social learning to the proximate factor of uncertainty [36]: narrow landscapes that provide little feedback in flat areas are likely to provoke uncertainty, and therefore, increase reliance on social learning.

Our aim in this study is to test these modelling predictions concerning peak width experimentally using the virtual arrowhead task, which in all previous studies has employed relatively wide peaks that offer reliable feedback to individual learners (figure 1, blue line). Therefore, we compared learning within this wide-peaked environment to a novel narrow-peaked search landscape condition (figure 1, red line), in which the same attributes are associated with the same bimodal search landscape, but with narrower optimal peaks. We tested three hypotheses:

H1: Individual learning is more difficult in the narrow condition, where peaks are more difficult to find (prediction: pure individual learners will perform worse in the narrow condition than in the wide condition);

H2: Social learning provides a solution to this, as social learners can learn the location of hard-to-find peaks from others (prediction: social learners will do equally well in both wide and narrow conditions, given that in both conditions they can copy equally matched demonstrators, one of whom has discovered the globally optimal peak);

H3: Social learning should be more useful in the narrow condition because individual learning is more difficult (prediction: participants will copy more often in the narrow condition than in the wide condition).

Note that in order to test H2 properly, we need to ensure that demonstrator performance is matched across the two conditions (narrow and wide peaks), such that in both conditions participants could potentially copy similarly high-scoring demonstrators. Otherwise, differences in performance could simply arise from participants in the wide condition having higher scoring demonstrators to copy than participants in the narrow condition. This would confound our intended manipulation: the landscape-generated difficulty of individual learning experienced by social learners. Therefore, we used artificially generated demonstrators in both conditions such that demonstrator performance was roughly matched (see Demonstrators section below). This ensured that the only difference between the two conditions was the difficulty of individual learning (more difficult in the narrow-peaked condition, assuming H1 is supported), and not differences in demonstrator quality.

## 2. Material and methods

### 2.1. Task

In the computer-based virtual arrowhead task participants engage in virtual ‘hunts’ where they accumulate a score based on the attributes of their arrowhead. The arrowhead has five attributes. Two of them—shape and colour—are discrete, taking one of four unordered values. Three of them—height, width and thickness—are continuous, and can take values ranging from 1 to 100 arbitrary units.

The score on each hunt is the weighted sum of four functions that convert four of the attribute values into pay-offs (colour is neutral, and has no effect on score). Shape has a step function and was identical across all conditions, so is not considered further. Of particular importance are the three continuous attributes, each of which is associated with a bimodal function (figure 1), creating a multimodal search landscape. The highest peak gives participants a hunt score of 1000 virtual ‘calories’. Finally, a small, normally distributed, positive or negative random value is added to the score, in order to simulate stochastic feedback from the environment. On each hunt, participants can freely modify all the attributes of their arrowhead, and they receive direct feedback of their score after the hunt.

After five practice hunts, participants engaged in three hunting seasons, each composed of 30 hunts. At the start of each season, the search landscape is reinitialized, i.e. optimal peaks are moved to different values of the attributes, thus simulating a form of environmental variability. Optimal peaks are not changed with the seasons. Participants are (accurately) informed that there is between-season but not within-season environmental variation.

### 2.2. Design

We manipulated two independent variables in a 2×2 design: learning (individual-only or individual-plus-social), and peak width (wide or narrow). In the individual-learning-only (henceforth ‘individual learning’) condition, participants could modify attributes on each hunt, receive feedback from the hunt, and try, over successive hunts, to reach the highest possible cumulative score. In the individual-plus-social-learning (henceforth ‘social learning’) condition, on each hunt participants could choose to use individual learning as in the individual learning condition, or they could choose to select one of five demonstrators to copy. These demonstrators are shown on the screen alongside each demonstrators’ cumulative scores, allowing participants to preferentially select the highest-scoring demonstrator (‘success-biased’ social learning).

In the wide condition, the bimodal function for the three continuous attributes generates peaks with a standard deviation of the normal distribution of *σ*=0.025. In the narrow condition, the same function is used, but with a smaller standard deviation of *σ*=0.01 which generates narrower peaks. One problem here is that this automatically inflates scores in the wide condition, as there is a larger total area under the wide-peaked bimodal functions than the narrow-peaked functions. Therefore, to keep the overall score comparable across the two conditions, in the narrow condition all scores below 560 ‘calories’ were set to 560, ensuring that the area below the two curves was the same (figure 1).

### 2.3. Participants

Eighty participants (57 female, age range 18–39, mean age=21.73) completed the experiment, all were students of the University of Birmingham, UK. Twenty participants were randomly assigned to the individual learning condition, with 10 in the wide and 10 in the narrow condition. Sixty participants were randomly assigned to the social learning condition, with 30 in the wide and 30 in the narrow condition. Ethical approval was granted by the Ethical Review Committee of the University of Birmingham, UK (review ERN 14-0218). All participants received the same amount of course credit for participating, as well as additional monetary payment of £0.07 per calorie as a performance incentive. Participants could receive a maximum of 1000 calories per hunt, so at 30 hunts per season and three seasons gave a maximum payment of 90 000× £0.07 = £6.30. Most participants received about 60 000 calories which equalled £4.20 each.

### 2.4. Demonstrators

Participants in the social learning condition could copy one of five individual-learning-only demonstrators. To avoid large differences between wide and narrow conditions in demonstrator performance, which would confound any wide versus narrow comparison of social learners’ performance, participants in the social learning condition could copy from a pre-programmed set of demonstrators created artificially by us. These were matched by condition, i.e. narrow social learners could copy one of five narrow demonstrators, and wide social learners could copy one of five different wide demonstrators, because pay-offs were specific to fitness landscape (i.e. the same combination of attribute values gave different pay-offs in the two landscapes). The ten demonstrators were created by replicating the hill-climbing win-stay-lose-shift algorithm previously found to describe individual learning in this task [32]: (i) pick one continuous attribute at random (e.g. height) and get its pay-off, (ii) on the next hunt randomly either increase or decrease it by five units, (iii) if pay-off increases then keep increasing or decreasing, else switch to decreasing or increasing, (iv) repeat until a switch has occurred on three hunts in a row (indicating oscillation around a peak), (v) move to another attribute and repeat the whole procedure. Shape was modified by picking all four values and retaining the highest scoring one. Initial search values were chosen such that in both conditions, one demonstrator eventually found all four attributes’ global peaks (giving a maximum score of 1000 by the end of the season), one demonstrator found three global and one local, one found two global and two local, one found one global and three local, and one found all local peaks. Therefore, in both narrow and wide conditions socially learning participants could copy one demonstrator who would give them the maximum pay-off.

### 2.5. Procedure

Participants completed the experiment in groups of varying size, given that demonstrator data were pre-programmed into the task and participants were not learning from each other in real time. Each participant sat at a computer terminal and completed the entire task via standardized on-screen instructions. Participants were precluded from speaking to others or writing anything down during the session. Once the participant completed all three seasons, they received payment and were debriefed. The entire study took no longer than 1 h. The information sheet for participants, the consent form and the debriefing sheet are included in the electronic supplementary material.

## 3. Results

**Figure 2.**
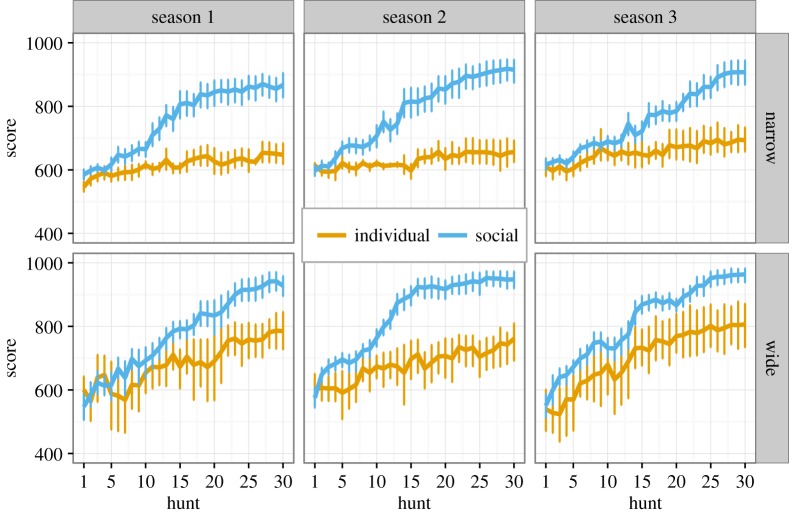
Performance (score in calories per hunt) over time (i.e. hunt) across the conditions and seasons. Scores started out at similar values, but diverged in the different conditions: individual learners performed better in the wide condition, while social learners performed similarly well in wide and narrow conditions. Error bars show 95% confidence intervals.

Performance (i.e. score per hunt in calories) increased over time for all participants, indicating that learning occurred, although the magnitude and rate of the increase varied across conditions (figure 2). A multilevel regression was run on score using package lme4 [37] in R [38] with scores nested within seasons, nested within participants. Original data (data.csv) and the script to reproduce the analysis (analysis.R) are included in the electronic supplementary material. Predictors were hunt, learning (individual versus social), peaks (narrow versus wide), age and sex (see electronic supplementary material, ‘Supplementary analyses’). The best fitting model had interactions between hunt and peaks, and between hunt and learning. Neither sex nor age had strong effects, nor were they predicted to, so we excluded them from subsequent analyses. The interactions with hunt emerged because of the improvement in score over the hunts: in all four conditions (individual learning+narrow, individual learning+wide, social learning+narrow and social learning+wide) participants started roughly with the same score, then differences emerged in later hunts between conditions. To address the three hypotheses we, therefore, looked just at the final scores on the last (30th) hunt of each season, both the final score obtained on that hunt (out of 1000) and the total cumulative score obtained at that hunt, i.e. the sum of all 30 hunts during a season, each one of which gave a maximum of 1000 calories, so out of 30 000. Season was included as a random effect.

### 3.1. Hypothesis H1: is individual learning more difficult in the narrow condition?

**Figure 3.**
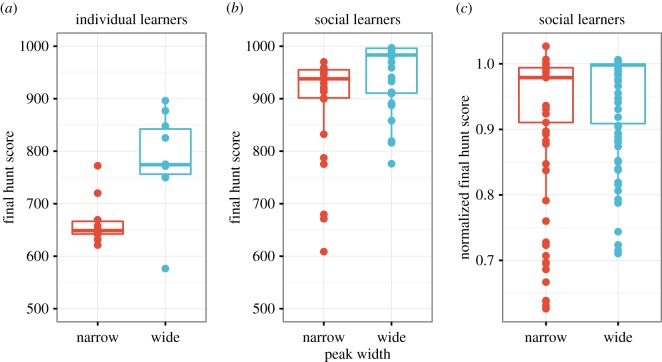
Difference in final hunt score between wide and narrow conditions in (*a*) individual learners, (*b*) social learners’ non-normalized raw scores and (*c*) social learners’ normalized scores to account for differences in demonstrator scores between the two conditions. Each point represents one participant’s mean score across all three seasons. Boxplots show medians and interquartile ranges, with whiskers extending to 1.5 IQR.

For both measures individual learners did better in the wide than in the narrow condition. Individual learners in the wide condition had scores on the final hunt that were 118.81 (s.e.=21.89, 95% CI [75.20, 162.41]) calories higher than those of individual learners in the narrow condition (figure 3*a*), and final cumulative scores that were 1667.60 (s.e.=466.90, 95% CI [737.70, 2597.61]) calories higher than those of individual learners in the narrow condition, with season as a random factor in both models. This supports hypothesis H1 that individual learning is more difficult in the narrow condition and confirms that our manipulation of peak width was successful.

### 3.2. Hypothesis H2: do social learners perform equally well in the wide and narrow conditions?

Looking at final hunt and cumulative scores (shown in figure 2), social learners performed slightly better in the wide than the narrow condition. Social learners in the wide condition had scores on the final hunt that were 49.94 (s.e.=13.67, 95% CI [23.01, 76.88]) calories higher than those of social learners in the narrow condition (figure 3*b*) and final cumulative scores that were 1333.60 (s.e.=261.20, 95% CI [818.86, 1848.41]) calories higher than those of social learners in the narrow condition. On the basis of this comparison, we must reject H2, and conclude that while social learning is of great help such that the difference between narrow and wide conditions is much smaller for social learners than individual learners (cf. figure 3*a*,*b*), social learning does not allow social learners in the narrow condition to fully match the performance of social learners in the wide condition.

However, despite artificially creating demonstrators that were matched for performance across the narrow and wide conditions, there were unavoidable differences between demonstrator scores across the two conditions (see electronic supplementary material, ‘Supplementary analyses’). This is particularly the case for final cumulative scores given that search in the wide landscape will accrue more calories during the hill-climbing than search in the narrow condition, where this occurs mostly on a flat landscape.

Therefore, we normalized the social learners’ final hunt and final cumulative scores by dividing the participants’ scores by the best demonstrator’s score in their condition. A normalized score of 1 indicates identical performance to the best demonstrator, and scores less than 1 indicate worse performance. Regression models with these normalized scores indicate that normalizing for demonstrator scores removes much of the difference found for the raw scores, such that 95% CIs for normalized scores overlapped with zero for both final hunt score (*b*=0.021, s.e.=0.014, 95% CI [−0.007, 0.049], figure 3*c*) and final cumulative score (*b*=0.007, s.e.=0.010, 95% CI [−0.013, 0.027]). This supports hypothesis H2 that social learners perform equally well in the narrow and wide conditions, after controlling for differences in demonstrator performance.

Additional analyses showed that social learners outperformed individual learners in both the wide and narrow conditions, as expected given previous studies using this task. In the narrow condition, social learners had 231.09 (s.e.=20.14, 95% CI [191.29, 270.88]) more calories in the final hunt than individual learners, and their cumulative score was 4025.60 (s.e.=365.00, 95% CI [3305.07, 4746.93]) calories higher than individual learners. In the wide condition, social learners had 162.22 (s.e.=17.86, 95% CI [126.93, 197.52]) more calories in the final hunt than individual learners and their cumulative scores were 3691.60 (s.e.=386.10, 95% CI [2928.62, 4454.49]) calories higher than individual learners. Hence social learners outperformed individual learners in both conditions, but to a greater extent in the narrow condition.

### 3.3. Hypothesis H3: do social learners copy more in the narrow than the wide condition?

**Figure 4.**
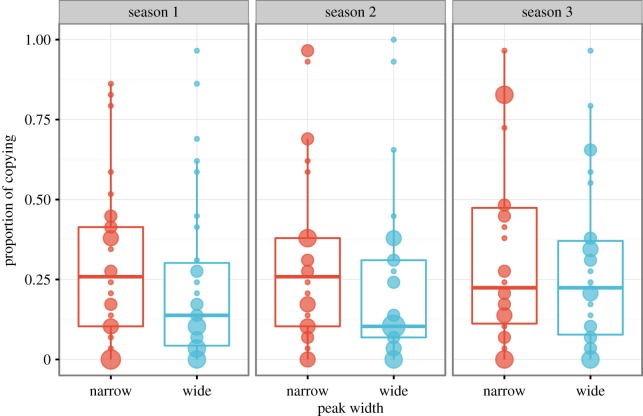
Comparison of copying frequency in the narrow and wide conditions, across the three seasons. The value shown is the proportion of hunts on which participants chose to copy, from 0 (never copied) to 1 (always copied). The size of the circles are proportional to the number of participants at that frequency. Boxplots show medians and interquartile ranges, with whiskers extending to 1.5 IQR.

Averaging across seasons and participants, the proportion of hunts (ranging from 0 to 1) on which social learners copied in the narrow condition was 0.31 (s.d.=0.26), and in the wide condition was 0.25 (s.d.=0.22), as shown in figure 4. Although this was in the predicted direction, there was large variation across participants in frequency of copying as indicated by the large standard deviations and large data spread shown in figure 4. Accordingly, a non-parametric Wilcoxon’s test (given that copying frequency was non-normally distributed) showed that the difference between narrow and wide copying frequencies was not significant overall (*W*=513.5, *p*=0.35), nor for each season separately (Season 1: *W*=513, *p*=0.35; Season 2: *W*=552.5, *p*=0.13; Season 3: *W*=482.5, *p*=0.64) An alternative approach is to use quasi-binomial regression, which allows for under-dispersed count data (as there were several participants who never or seldom copied). Quasi-binomial regression on the mean copying frequency across all three seasons similarly showed no difference in copying frequency between wide and narrow condition (*β*=0.32, s.e.=0.31, 95% CI [−0.93, 0.28]). So while there was a trend for more copying in the narrow condition than the wide condition, particularly during seasons 1 and 2 (figure 4), the difference was not significant, so hypothesis H3 is not supported.

**Figure 5.**
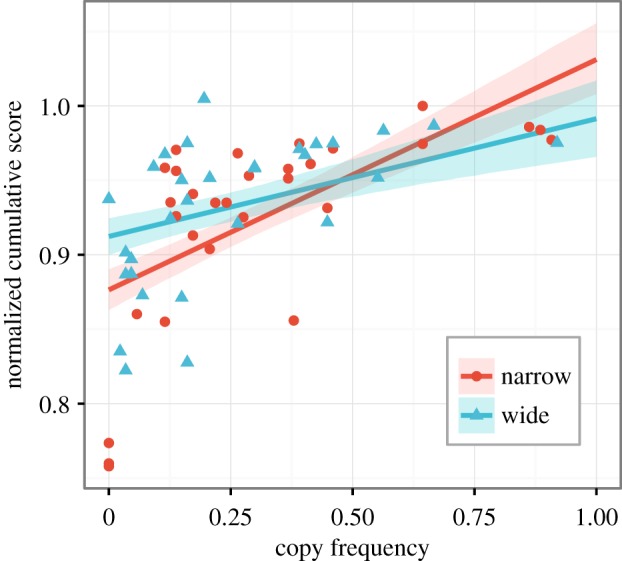
Relationship between copying frequency and final normalized cumulative score across social learners in the wide and narrow conditions. Lines are best-fit multilevel regression lines with season as a random factor. Shaded areas show 80% prediction intervals calculated using the predictInterval function from package merTools [39].

The fact that social learners as a group outperformed individual learners (figure 3) shows that social learning is beneficial, but we can also ask whether there is a relationship at the participant level between copying frequency and performance. Multilevel regressions with season as a random factor show that copy frequency significantly predicts final normalized, cumulative score in both the wide (*β*=0.079, s.e.=0.024, 95% CI [0.031, 0.126]) and the narrow (*β*=0.155, s.e.=0.024, 95% CI [0.109, 0.202]) conditions (figure 5). The regression slope in the narrow condition is roughly twice as large as in the wide condition, indicating that copying was more beneficial in the narrow condition than the wide condition.

In electronic supplementary material, ‘Supplementary analyses’, we present additional analyses to show that there are no demonstrable differences in the frequency distributions of copying across the two conditions (e.g. it is not the case that there are more participants who never copied in the wide condition than in the narrow condition), and that there is no difference in the timing of copying (e.g. participants in the narrow condition do not copy earlier than participants in the wide condition).

## 4. Conclusion

The aim of this study was to explore experimentally how varying the smoothness of the cultural fitness landscape affects the adaptiveness of, and people’s use of, social and individual learning. Previous models [34,35] found that social learning is more useful when search landscapes contain narrow fitness peaks. This is because narrow-peaked landscapes make individual learning more difficult. In narrow-peaked landscapes, guesses or partial solutions that fall within flat regions of the fitness landscape provide little or no information on how close one is to a locally or globally optimal peak. Social learning circumvents this by allowing individuals to copy hard-to-discover solutions from others. In wide-peaked landscapes, by contrast, individual learning is more straightforward. Simple hill-climbing algorithms can reach optima with little or no social learning needed. Here we tested this idea experimentally using a task in which participants design artefacts, improve them via individual and/or social learning and receive pay-offs based on a multimodal fitness landscape.

As predicted in hypothesis H1, individual learning was more difficult in the narrow-peaked environment than in the wide-peaked environment, as demonstrated by the fact that our individual-learning-only participants performed much worse in the former condition (figure 3*a*). Hypothesis H2, that social learners should perform equally well in both conditions, was partially supported. Looking at the raw scores (figure 3*b*), social learners in the narrow condition performed much better relative to individual learners in the narrow condition, but still not quite at the level of social learners in the wide condition. This shows that social learning greatly ameliorates the difficulty of individual learning in narrow-peaked environments, but not perfectly. Some of this shortcoming, however, was due to unavoidable differences in demonstrator quality; when correcting for this, the difference in score between narrow and wide social learners virtually disappeared (figure 3*c*). Effectively, social learning acted as a buffer, resulting in higher pay-offs irrespective of the difficulty of the task being faced.

Previous studies with this task have shown that one advantage of social learning relative to pure individual learning is that social learning allows individual learners to escape suboptimal peaks within a multimodal fitness landscape [29,30]. This effect was replicated here, given that social learners out-performed individual learners even in the wide condition. The novelty of our findings here is in showing that this effect is magnified when peaks are narrowed, making them more difficult to find via individual learning alone [34,35].

While social learners in the narrow condition showed a trend for copying more often in the narrow condition, as predicted in hypothesis H3, this difference was not large, and swamped by individual variation in copying frequency (figure 4). As in previous studies using this and other tasks [18,21,23–25], even though social learning resulted in higher pay-offs in the game, and higher monetary rewards in the real world, few participants copied often. How can we explain this anomaly? One possibility is that frequency of copying is not an appropriate measure: perhaps, only a few copying episodes are needed to locate a hard-to-find peak in the narrow condition, and so copying was more of an all-or-nothing behaviour rather than a continuous one (i.e. copying leads to higher scores, but *more* copying does not equal higher scores). However, the finding that copying frequency significantly predicted score suggests against this: more copying *did* lead to higher scores in our experiments. However, it might be possible that the advantage of copying was not enough to warrant participants increasing their copying rate. The difference between pure individual learning (copy frequency=0) and pure social learning (copy frequency=1) is not very large (see e.g. the slopes in figure 5) and, in our experiment, equals approximately 2500 calories per season, or 50 pence in total. Future studies could explore whether increasing the incentives would increase the frequency of social learning.

Another possibility is that individuals just did not realize that individual learning was in fact more difficult in the narrow-peak condition. However, if this were the case, then we would expect copying to increase, even slightly, in seasons 2 and 3, compared with season 1, as participants realize that individual learning is less effective. Yet, this was not the case (see electronic supplementary material, ‘Supplementary analyses’). Finally, it is possible our Western participants possess over-inflated tendencies, or pre-existing norms, to engage in individual learning. A recent study, using the same task as employed here, showing higher frequencies of social learning in participants from mainland China supports this interpretation [31], and further cross-cultural research is needed to explore this possibility.

The distinction between narrow- and wide-peaked search landscapes represents, in our opinion, an important addition to the experimental and theoretical literature on the evolutionary basis of social learning. Several current debates can be reframed and clarified in similar terms. For example, the view that human cultural transmission is characterized by low-fidelity transmission processes, guided by the presence of ‘attractors’ that ensure that most individuals converge on the same, or similar, end results, in opposition to high-fidelity transmission processes ([40–42], see a review in [33]) can be interpreted as a ‘wide-peaked landscape’ view of culture. While this might be the case for some cultural domains (e.g. supernatural representations, folk tale themes or aesthetic preferences), other domains, for example, those related to complex technologies, are likely to be represented by narrow-peaked search spaces, and to require individuals to use high-fidelity social learning in order to find the correct solutions. An analogous logic can be applied to the differences between the culture of humans and non-human animals. In non-human species, ‘cultural’ behaviours are likely to be represented by wide-peaked search spaces, i.e. behaviours that can be reproducible with individual learning helped by low-fidelity forms of social learning (‘zone of latent solutions’ in [7]). We think it likely that during our evolution hominins started to invent effective solutions to problems in the form of narrow-peaked spaces, for which effective social learning skills were needed [34].

In conclusion, we suggest that attention to tasks’ design space is important both for experiments and models aiming to investigate the adaptiveness of social and individual learning. In this study, we made a first step in this direction, showing how narrow- and wide-peaked search spaces can influence the outcomes of humans’ learning behaviour.

## Supplementary Material

ESM.pdf: combined pdf with Supplementary Analyses, The Information sheet for participants, the Consent Form, and the Debriefing Sheet.

## Supplementary Material

analysis.R: script to perform the analysis described in the manuscript.

## Supplementary Material

data.csv: raw data.

## Data accessibility

All data and R analysis scripts are included as the electronic supplementary material.

## References

[1] Heyes CM 1994 Social learning in animals: categories and mechanisms. *Biol. Rev. Camb. Philos. Soc.* 69, 207–231. (doi:10.1111/j.1469-185X.1994.tb01506.x) 8054445 10.1111/j.1469-185x.1994.tb01506.x

[2] Hoppitt W, Laland KN 2013 *Social learning: an introduction to mechanisms, methods, and models*. Princeton, NJ: Princeton University Press.

[3] Mesoudi A 2011 *Cultural evolution*. Chicago, IL: University of Chicago Press.

[4] Henrich J 2015 *The secret of our success: how culture is driving human evolution, domesticating our species, and making us smarter*. Princeton, NJ: Princeton University Press.

[5] Richerson PJ, Boyd R 2005 *Not by genes alone: how culture transformed human evolution*. Chicago, IL: University of Chicago Press.

[6] Dean LG, Kendal RL, Schapiro SJ, Thierry B, Laland KN 2012 Identification of the social and cognitive processes underlying human cumulative culture. *Science* 335, 1114–1118. (doi:10.1126/science.1213969) 22383851 10.1126/science.1213969 PMC4676561

[7] Tennie C, Call J, Tomasello M 2009 Ratcheting up the ratchet: on the evolution of cumulative culture. *Phil. Trans. R. Soc. B* 364, 2405–2415. (doi:10.1098/rstb.2009.0052) 19620111 10.1098/rstb.2009.0052 PMC2865079

[8] Laland KN 2004 Social learning strategies. *Learn. Behav.* 32, 4–14. (doi:10.3758/BF03196002) 15161136 10.3758/bf03196002

[9] Rendell L, Fogarty L, Hoppitt WJE, Morgan TJH, Webster MM, Laland KN 2011 Cognitive culture: theoretical and empirical insights into social learning strategies. *Trends Cognit. Sci.* 15, 68–76. (doi:10.1016/j.tics.2010.12.002) 21215677 10.1016/j.tics.2010.12.002

[10] Boyd R, Richerson PJ 1985 *Culture and the evolutionary process*. Chicago, IL: University of Chicago Press.

[11] Henrich J, McElreath R 2003 The evolution of cultural evolution. *Evol. Anthropol.* 12, 123–135. (doi:10.1002/evan.10110)

[12] Rogers AR 1988 Does biology constrain culture. *Am. Anthropol.* 90, 819–831. (doi:10.1525/aa.1988.90.4.02a00030)

[13] Boyd R, Richerson PJ 1995 Why does culture increase human adaptability? *Ethol. Sociobiol.* 16, 125–143. (doi:10.1016/0162-3095(94)00073-G)

[14] Aoki K, Wakano JY, Feldman MW 2005 The emergence of social learning in a temporally changing environment: a theoretical model. *Curr. Anthropol.* 46, 334–340. (doi:10.1086/428791)

[15] Enquist M, Eriksson K, Ghirlanda S 2007 Critical social learning: a solution to Rogers’s paradox of nonadaptive culture. *Am. Anthropol.* 109, 727–734. (doi:10.1525/aa.2007.109.4.727)

[16] Henrich J, Boyd R 1998 The evolution of conformist transmission and the emergence of between-group differences. *Evol. Hum. Behav.* 19, 215–241. (doi:10.1016/S1090-5138(98)00018-X)

[17] Morgan TJH, Laland KN 2012 The biological bases of conformity. *Front. Neurosci.* 6, 87 (doi:10.3389/fnins.2012.00087) 22712006 10.3389/fnins.2012.00087 PMC3375089

[18] McElreath R, Lubell M, Richerson PJ, Waring TM, Baum W, Edsten E, Efferson C, Paciotti B 2005 Applying evolutionary models to the laboratory study of social learning. *Evol. Hum. Behav.* 26, 483–508. (doi:10.1016/j.evolhumbehav.2005.04.003)

[19] Toelch U, van Delft MJ, Bruce MJ, Donders R, Meeus MTH, Reader SM 2009 Decreased environmental variability induces a bias for social information use in humans. *Evol. Hum. Behav.* 30, 32–40. (doi:10.1016/j.evolhumbehav.2008.07.003)

[20] McElreath R, Bell AV, Efferson C, Lubell M, Richerson PJ, Waring T 2008 Beyond existence and aiming outside the laboratory: estimating frequency-dependent and pay-off-biased social learning strategies. *Phil. Trans. R. Soc. B* 363, 3515–3528. (doi:10.1098/rstb.2008.0131) 18799416 10.1098/rstb.2008.0131 PMC2607339

[21] Mesoudi A 2011 An experimental comparison of human social learning strategies: payoff-biased social learning is adaptive but underused. *Evol. Hum. Behav.* 32, 334–342. (doi:10.1016/j.evolhumbehav.2010.12.001)

[22] Muthukrishna M, Morgan T, Henrich J 2015 The when and who of social learning and conformist transmission. *Evol. Hum. Behav.* 37, 10–20. (doi:10.1016/j.evolhumbehav.2015.05.004)

[23] Toelch U, Bruce MJ, Newson L, Richerson PJ, Reader SM 2014 Individual consistency and flexibility in human social information use. *Proc. R. Soc. B* 281, 20132864 (doi:10.1098/rspb.2013.2864) 10.1098/rspb.2013.2864 PMC3871325 24352950

[24] Toelch U, Bach DR, Dolan RJ 2014 The neural underpinnings of an optimal exploitation of social information under uncertainty. *Soc. Cognit. Affect. Neurosci.* 9, 1746–1753. (doi:10.1093/scan/nst173) 24194580 10.1093/scan/nst173 PMC4221218

[25] Efferson C, Lalive R, Richerson P, McElreath R, Lubell M 2008 Conformists and mavericks: the empirics of frequency-dependent cultural transmission. *Evol. Hum. Behav.* 29, 56–64. (doi:10.1016/j.evolhumbehav.2007.08.003)

[26] Arthur WB 2009 *The nature of technology: what it is and how it evolves*. New York, NY: Simon and Schuster.

[27] Boyd R, Richerson PJ 1992 How microevolutionary processes give rise to history. In *Evolution and history* (ed. M Nitecki), pp. 179–209. Chicago, IL: University of Chicago Press.

[28] Wright S 1932 The roles of mutations, inbreeding, crossbreeding and selection in evolution. In *Proc. of the 11th Int. Congress of Genetics*, vol. 1. pp. 356–366. Brooklyn, NY: Brooklyn Botanic Garden.

[29] Mesoudi A, O’Brien M 2008 The cultural transmission of great basin projectile-point technology I: an experimental simulation. *Am. Antiq.* 73, 3–28. See http://www.jstor.org/stable/25470456.

[30] Mesoudi A 2008 An experimental simulation of the ‘copy-successful-individuals’ cultural learning strategy: adaptive landscapes, producer-scrounger dynamics, and informational access costs. *Evol. Hum. Behav.* 29, 350–363. (doi:10.1016/j.evolhumbehav.2008.04.005)

[31] Mesoudi A, Chang L, Murray K, Lu HJ 2015 Higher frequency of social learning in China than in the West shows cultural variation in the dynamics of cultural evolution. *Proc. R. Soc. B* 282, 1–7. (doi:10.1098/rspb.2014.2209) 10.1098/rspb.2014.2209 PMC4262178 25392473

[32] Mesoudi A, O’Brien M 2008 The cultural transmission of great basin projectile-point technology II: an agent-based computer simulation. *Am. Archaeol.* 73, 627–644. See http://www.jstor.org/stable/25470521.

[33] Acerbi A, Mesoudi A 2015 If we are all cultural Darwinians what’s the fuss about? clarifying recent disagreements in the field of cultural evolution. *Biol. Philos.* 30, 481–503. (doi:10.1007/s10539-015-9490-2) 26085703 10.1007/s10539-015-9490-2 PMC4461798

[34] Acerbi A, Tennie C, Nunn CL 2011 Modeling imitation and emulation in constrained search spaces. *Learn. Behav.* 39, 104–114. (doi:10.3758/s13420-010-0009-z) 21264559 10.3758/s13420-010-0009-z

[35] Acerbi A, Jacquet PO, Tennie C 2012 Behavioral constraints and the evolution of faithful social learning. *Curr. Zool.* 58, 307–318. (doi:10.1093/czoolo/58.2.307)

[36] Morgan TJH, Rendell LE, Ehn M, Hoppitt W, Laland KN 2012 The evolutionary basis of human social learning. *Proc. R. Soc.* 279, 653–662. (doi:10.1098/rspb.2011.1172) 10.1098/rspb.2011.1172 PMC3248730 21795267

[37] Bates D, Mächler M, Bolker B, Walker S 2015 Fitting linear mixed-effects models using lme4. *J. Stat. Softw.* 67, 1–48. (doi:10.18637/jss.v067.i01)

[38] R Core Team. 2016 R: a language and environment for statistical computing. Vienna, Austria: R Foundation for Statistical Computing.

[39] Knowles JE, Frederick C 2016 merTools: Tools for analyzing mixed effect regression models [R package version 0.2.1]. See https://CRAN.R- project.org/package=merTools.

[40] Sperber D 1996 *Explaining culture: a naturalistic approach*. New York, NY: Wiley.

[41] Claidière N, Scott-Phillips TC, Sperber D 2014 How Darwinian is cultural evolution? *Phil. Trans. R. Soc. B* 369, 20130368 (doi:10.1098/rstb.2013.0368) 24686939 10.1098/rstb.2013.0368 PMC3982669

[42] Morin O 2015 *How traditions live and die*. Oxford, UK: Oxford University Press.

