# Supplementary material for: Social learning solves the problem of narrow-peaked search landscapes: experimental evidence in humans
Source: R Soc Open Sci. 2016 Sep 7;3(9):160215. doi: 10.1098/rsos.160215 (PMC5043306; doi:10.1098/rsos.160215)
Supplement: ESM.pdf: combined pdf with Supplementary Analyses, The Information sheet for participants, the Consent Form, and the Debriefing Sheet. [file rsos160215supp1.pdf]

Supplementary Analysis for
Acerbi, Tennie & Mesoudi
"Social learning solves the problem of narrow-peaked search landscapes: experimental evidence in humans"

All of the following analyses, as well as those reported in the main paper, can be re-run using the R script ("analysis.R") and data file ("data.csv") included as Electronic Supplementary Material.

1. Overall regression model for score

Multi-level regressions were run on score (i.e. performance/success in the task) using *lmer* in R, with scores nested within seasons nested within participants. The dependent variable is score per hunt (from 0-1000), the predictors are (i) hunt, (ii) learning (individual learning vs social), (iii) peaks (wide vs narrow), (iv) sex, and (v) age. The best-fitting model (Tables S1 and S2) has interactions between hunt and peaks, and between hunt and learning. Neither sex nor age have strong effects, nor were they predicted to, so we exclude them from subsequent analyses. Figure 2 in the paper shows how peaks and learning affect score.

Interactions with hunt emerge because of the improvement in score over the hunts: all conditions (narrow individual learning, narrow social learning, wide individual learning and wide social learning) start off roughly with the same score, then differences emerge in later hunts between peaks, and between learning conditions. We examine below exactly where these effects lie, and how strong they are. The lack of an interaction between peaks and learning suggests that social learners do not do better than individual learners in the narrow condition than in the wide condition as expected, although again this is examined later.

Table S1: Model comparison for multilevel regressions. All models have random slopes for hunt (given different slopes shown in Figure S1) and observations are nested within seasons within participants.

Model	Df	AIC	BIC	Log Likelihood	deviance	Chi	Df	p
Null	8	82273	82328	41128	82257			
Hunt	9	82134	82195	41058	82116	141.17	1	<0.001
Hunt + peaks + learning	11	82114	82190	41046	82092	23.20	2	<0.001
Hunt * peaks + learning	12	82110	82193	41043	82092	6.14	1	0.013
Hunt * learning + peaks	12	82071	82154	41024	82047	39.01	0	<0.001
Hunt * learning + hunt * peak	13	82061	82151	41018	82035	11.90	1	<0.001
Hunt * peak * learning	15	82061	82164	41015	82031	4.40	2	0.107

Table S2: Best fitting multi-level regression model predicting score, with scores nested within seasons nested within participants. Model is score ~ hunt * learning + hunt * peaks + (hunt | participant / season). Reference category for learning is individual, for peaks is narrow.

	Beta	SE	t value
(Intercept)	577.75	9.59	60.24
Hunt	3.81	0.83	4.60
Learning (social)	17.09	9.91	1.72
Peaks (wide)	7.78	8.58	0.91
Hunt * learning (social)	7.20	0.86	8.41
Hunt * peaks (wide)	2.56	0.74	3.46

2. Does performance over time increase?
Yes, but at different rates in the different groups/environments.

The significance of hunt in the overall regression suggests that score improves with hunt. This can also be seen in Figure 2, which shows different conditions having different rates of increase (e.g. individual learners improve very little compared to social learners, especially in the narrow condition). We can confirm this by calculating the regression slopes for hunt in each of the four conditions separately (Table S3). Individual learners in the narrow condition have the smallest increase over time, smaller than individual learners in the wide condition, as indicated by non-overlapping confidence intervals. Social learners learn faster, as expected, than individual learners, although the wide confidence intervals in the individual/wide condition overlap slightly with the social/narrow condition, indicating that some individual learners did well in the wide condition. Social learners increased in score faster in the wide condition than the narrow condition, although again with slightly overlapping confidence intervals. Overall, however, we can see that learning is faster when social learning is allowed, and in the wide peak environment.

Table S3: Regression slopes indicating improvement of score over hunts

Condition	Hunt coefficient	Standard error	95% CI
Individual / narrow	2.67	0.64	[1.28, 4.06]
Individual / wide	7.51	1.31	[4.82, 10.20]
Social / narrow	11.39	0.71	[9.95, 12.83]
Social / wide	13.19	0.47	[12.24, 14.15]

3. Is individual learning easier in the wide condition?
Yes. We can remove the effect of hunt by looking just at the final scores on hunt 30, both the final score obtained on hunt 30 itself (out of 1000) and the total cumulative score obtained at hunt 30 (the sum of all 30 hunts, so out of 30000), with season as a random effect.

For both measures individual learners did better in the wide condition. Individual learners in the wide condition have final cumulative scores that are 1667.60 (se=466.90, 95% CI [737.70, 2597.61]) calories higher than those of individual learners in the narrow condition (Figure S1). Individual learners in the wide condition have final hunt 30 scores that are 118.81 (se=21.89, 95% CI [75.20, 162.41]) calories higher than those of individual learners in the narrow condition (Figure

S2).

This confirms Hypothesis H1 that individual learning is significantly more difficult in the narrow condition, and confirms that our experimental manipulation was successful.

Figure S1: Difference in final cumulative score between wide and narrow individual learners

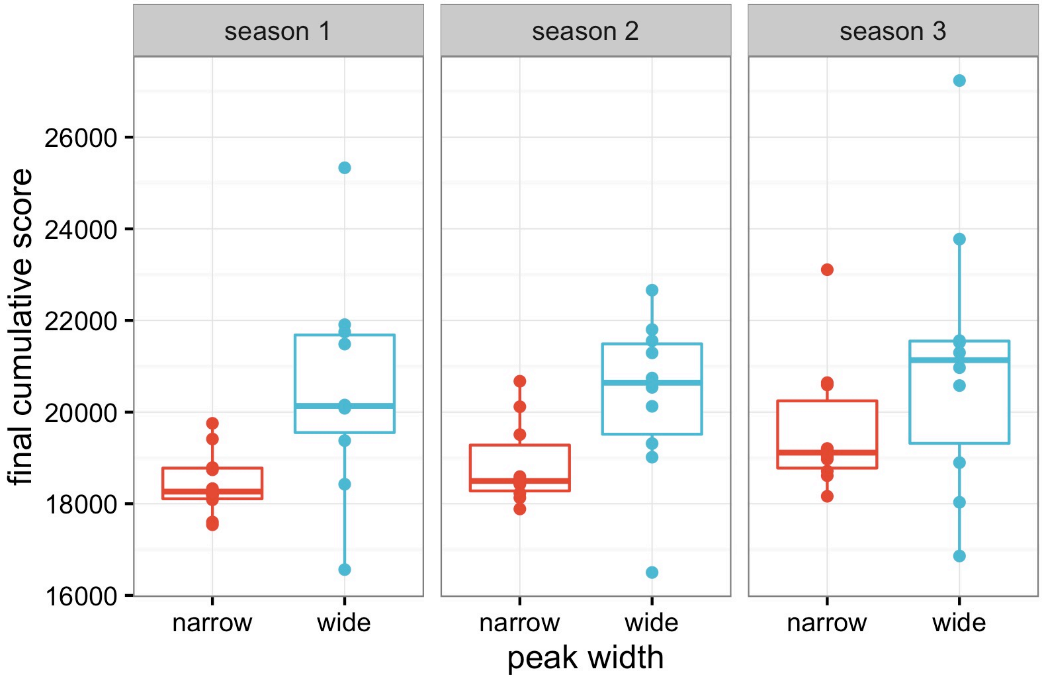

Figure S2: Difference in final kills between wide and narrow individual learners

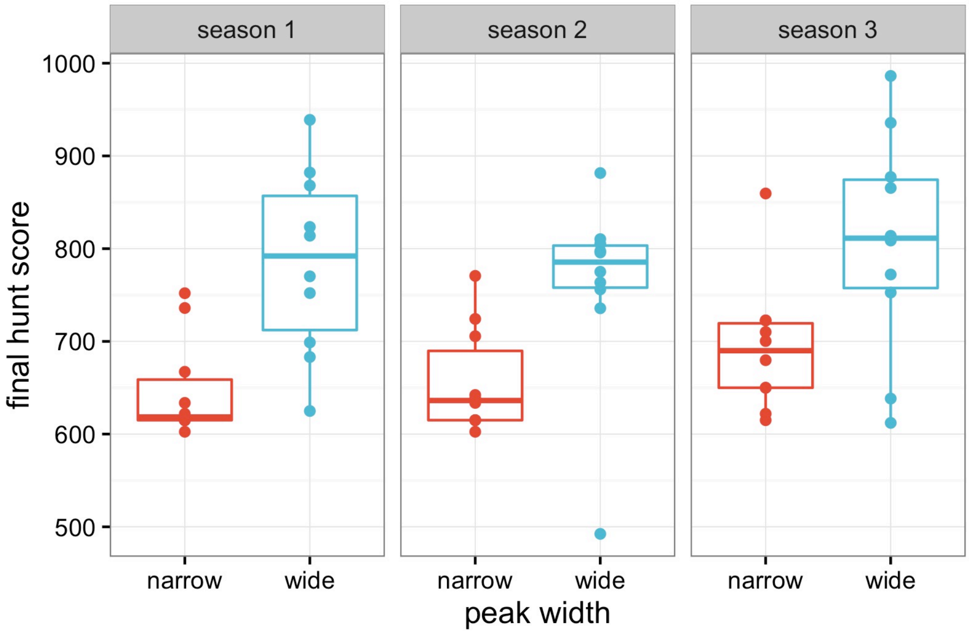

4. Do social learners out-perform individual learners?
Yes. Similar analyses show that social learners outperform individual learners in both conditions (Figures S3 and S4).

In the narrow condition, social learners have 231.09 (se=20.14, 95% CI [191.29, 270.88]) more calories in the final hunt than individual learners, and their cumulative score is 4025.60 (se=365.00, 95% CI [3305.07, 4746.93]) calories higher than individual learners.

In the wide condition, social learners have 162.22 (se=17.86, 95% CI [126.93, 197.52]) more calories in the final hunt than individual learners, and their cumulative score is 3691.60 (se=386.10, 95% CI [2928.62, 4454.49]) calories higher than individual learners.

Figure S3: Mean cumulative score of individual and social learners in the narrow and wide conditions

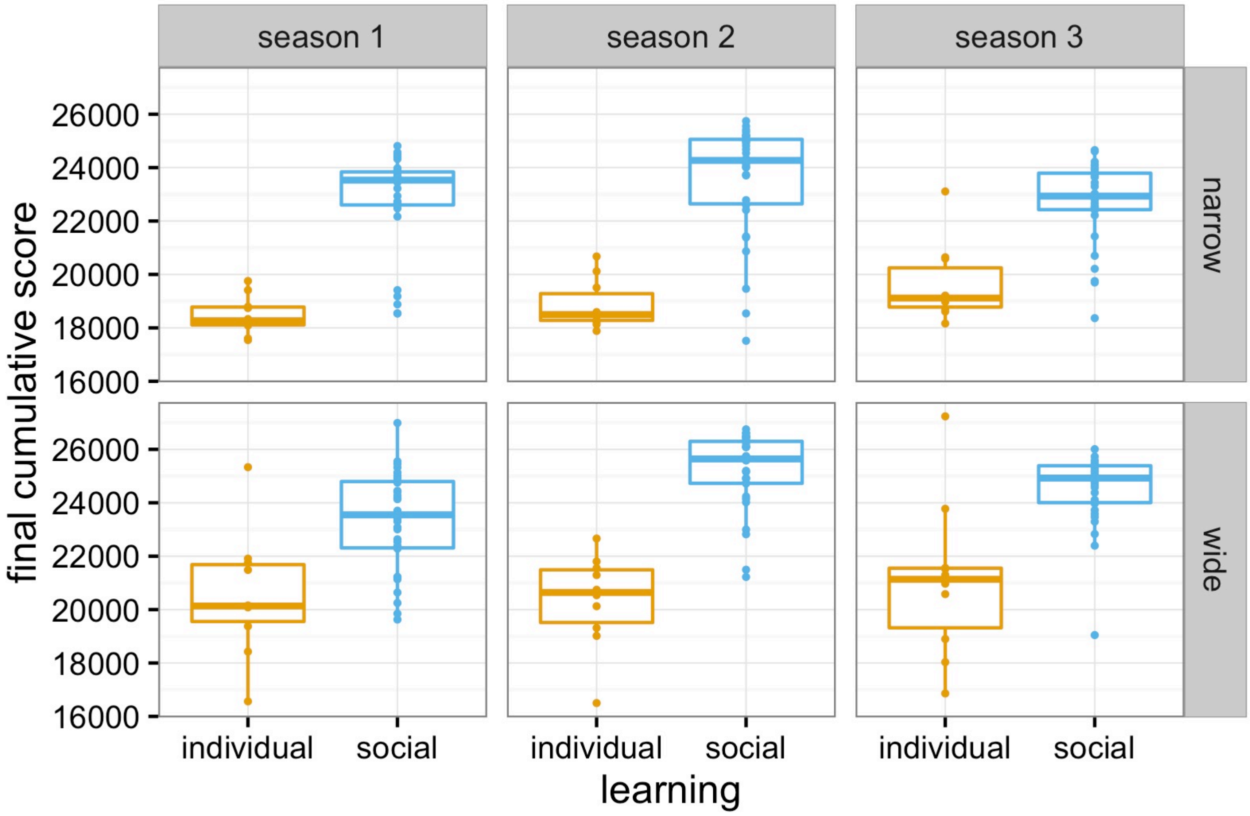

Figure S4: Mean final hunt score of individual and social learners in the narrow and wide conditions

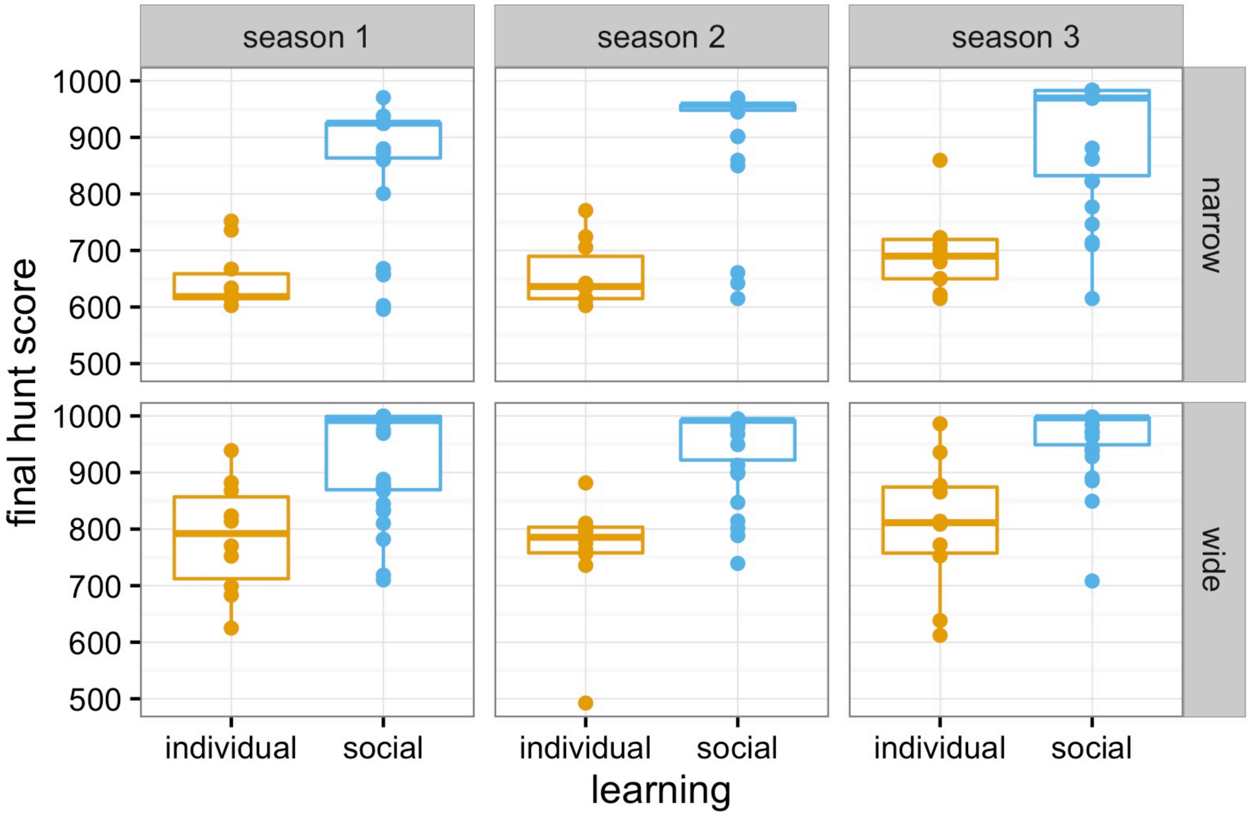

5. Do social learners do better in the wide condition than in the narrow condition?

Yes, but not when controlling for differences in demonstrator performance. Inspection of Figures S3 and S4 indicates that social learners do marginally better in the wide condition than the narrow condition, just like individual learners (see section 3). However, recall that narrow and wide social learners could copy from different sets of demonstrators matched to their environment (i.e. narrow social learners copied from narrow demonstrators, wide social learners copied from different wide demonstrators). When comparing narrow and wide social learners, we therefore need to account for differences in the scores of the best demonstrators in the respective groups (Table S4). While these were matched as much as possible during their creation, there are still some minor differences.

Table S4: differences in final cumulative score in the two sets of demonstrators

**Season**	**Best demonstrator's final cumulative score**		**Best demonstrator's final hunt score**	
	**Wide**	**Narrow**	**Wide**	**Narrow**
1	25525	24838	993	945
2	26728	25784	994	963
3	26101	24019	997	982

This can be done by normalising the social learners' scores, by dividing the participants' scores by the best demonstrator scores in their condition. A normalised score of 1 indicates identical performance to the best demonstrator, and scores less than 1 indicate worse performance.

Regression models with normalised scores indicate that social learners still score higher in the wide condition than the narrow condition but only marginally, and with confidence intervals overlapping zero, for both final hunt score (b = 0.021, se=0.014, 95% CI [-0.007, 0.049]) and final cumulative score (b = 0.007, se=0.010, 95% CI [-0.013, 0.027]).

6. Do social learners copy more often in the narrow condition?
Not convincingly. There was a trend for more copying in the narrow condition than the wide condition (Figure 4), but this difference was not significant. Copying frequency was non-normally distributed so we can use the non-parametric Wilcoxon test on the mean copying frequency across all seasons, which was not significant ($W=513.5$, $p<0.35$). Another approach is to use quasibinomial regression, which allows for under-dispersed count data (as we have when many people never copied). Quasibinomial regression on the mean copying frequency across all three seasons showed no difference in copying frequency (b = 0.32, se=0.31, 95% CI [-0.93, 0.28]).

7. Does copying more lead to higher scores?
Yes, in both wide and narrow conditions copy frequency positively predicts score, indicating that high copiers get higher scores.

Multi-level regressions with season as a random factor show that copy frequency (i.e. proportion of hunts on which the participant copied a demonstrator, ranging from 0 to 1) significantly predicts final normalised, cumulative score in both the wide (beta=0.079, se=0.024, 95% CI [0.031, 0.126]) and the narrow (beta=0.155, se=0.024, 95% CI [0.109, 0.202]) conditions. The regression slope in the narrow condition is roughly twice as large as in the wide condition, indicating that copying was relatively more beneficial in the narrow condition (see also Figure 5 in the main paper). This fits with the earlier observation in section 3 that learning in the narrow condition is more difficult than in the wide condition, but raises the puzzle of why participants did not therefore copy more, as shown in section 6.

8. How does individual variation in copying compare across the conditions?
The large data spread in Figure 4 suggest that there is large individual variation in copying frequency within each condition. Figure S5 shows the distribution of copying within the two groups, narrow and wide. Here we can see that the wide copiers peak at very low or zero copying frequencies, whereas narrow copiers are less likely to be zero-copiers. However, inspection of Table S5 indicates that these differences are not very strong, given the small sample size.

Figure S5: Individual variation in copying frequency

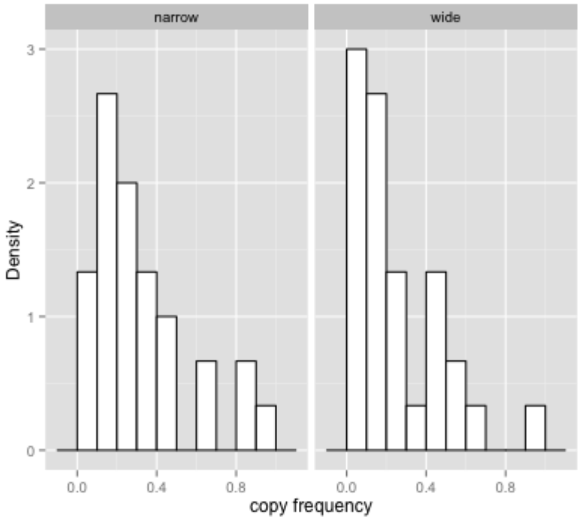

Table S5: Frequencies of copiers (mean across all seasons)

Proportion of hunts on which participant copied	narrow	wide
0	3	1
0.01 – 0.1	3	9
0.11 – 0.2	6	7
0.21 – 0.3	6	4
0.31 – 0.4	4	2
0.41 – 0.5	3	3
0.51 – 0.6	0	2
0.61 – 0.7	2	1
0.71 – 0.8	0	1
0.81 – 0.9	2	0
0.91 – 1	1	1

9. Do narrow copiers copy earlier than wide copiers?

Perhaps frequency of copying is not very informative in this situation, as only one or two copying events may be sufficient for a narrow copier to locate a hard-to-find peak. Subsequent copying would be unnecessary. Wide copiers, on the other hand, may prefer to copy later: they can easily find a peak via individual learning, but might use copying to subsequently locate a higher peak, if theirs is not the highest.

As shown in Figure S6, this does not appear to be the case: narrow copiers copy slightly more in the first hunt on which copying is possible (hunt 2), but this overlaps with wide copiers. Subsequently, narrow copying frequency remains slightly above the wide copying frequency, and always with overlapping error bars.

Figure S6: copying frequency per hunt, in narrow and wide social learners (NB copying is not permitted on the very first hunt)

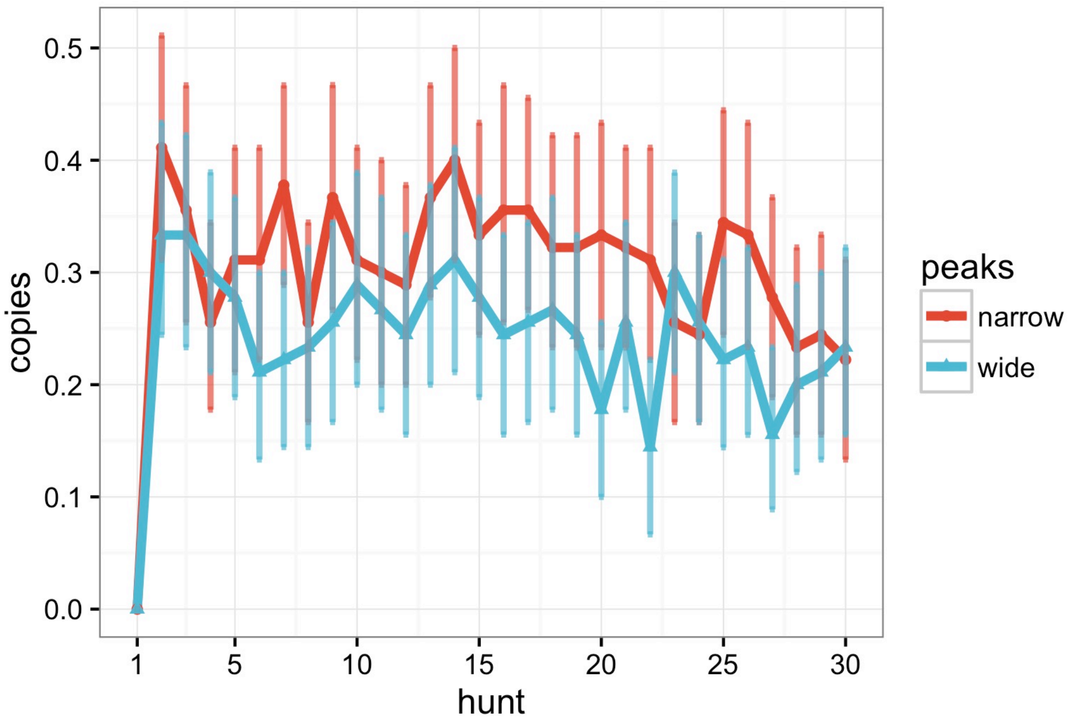

Research study “Virtual Arrowhead game”:
Information Sheet for participants

Principal Investigators: Dr. Claudio Tennie

Contact details: Dr Claudio Tennie - School of Psychology - University of Birmingham
Birmingham - B15 2TT - UK
Office: +44 (0)121 41 46795

Invitation to participate

We would like to invite you to be part of this research project, if you would like to. You should only agree to take part if you want to, it is entirely up to you. If you choose not to take part, there won’t be any disadvantages for you and you will hear no more about it.

Please read the following information carefully before you decide to take part; this will tell you why the research is being done and what you will be asked to do if you take part. Please ask if there is anything that is not clear or if you would like more information.

If you decide to take part you will be asked to sign the attached form to say that you agree.

You are still free to withdraw at any time without giving a reason. Your data will then be destroyed. You can contact the researchers with this request for 30 days after you have completed the study. The reason for this time limit is that the data is meant to be analysed for publication and once any articles or reports are published the researchers are unable to destroy your data (and 30 days after you have participated in the study the overall data will likely have been analysed and finalised/written up).

The data collected in this study will include the responses that you make on a computer task and basic demographic information about you (e.g., sex, ethnicity, age). The data collected in this study will be used only for the purpose described in this form, and will be available only to the principal investigator listed in the consent form and other personnel involved in this study at the University of Birmingham. Data gathered from this study will be maintained as long as required by regulations, which is up to 10 years following the publication of empirical articles or communications describing the results of the study.

Every effort will be taken to protect the names of the participants in this study. Your identity will not be recorded as part of your data, and will not be revealed in any publication that may result from this study; your consent form will not be stored with your data, to ensure that your identity cannot be linked in any way to your data. All information you provide will be kept confidential, except as governed by law.
In exchange for your participation, you will earn both credits and money (students) or money (all others). The task will take between 30 and 60 minutes to complete. According to time thus spent, you will thus earn between 0.5 and 1.0 credits toward your participation requirements. You will be able to earn up to £6.30 depending on your performance in the game.

There are no consequences for withdrawing, although the payment and credit received for your time and performance would be reduced to reflect the proportion of the study actually completed.

Study Details

This research study is a computer-based learning experiment. You will be asked to sit at a computer and engage in a task in which you have to design a 'virtual arrowhead' that is then used to perform 'virtual hunts'. You may be asked to choose to interact with other participants via the computer program.

The total length of the study will not exceed one hour and – if you are a current student of the University of Birmingham - you will be paid in credits as compensation for your time – depending on how long your test session lasts (1/2h = ½ credit, 1h = 1 credit etc.). Regardless of whether you are a current student, you will receive up to an additional £6.30 depending on your performance in the game. There are no foreseeable risks or discomforts involved. The data collected during this experiment are entirely anonymous and your name and contact details are not stored anywhere in our records. Anonymous, aggregate data (e.g. group averages) may be published as part of an article in a scientific journal, but you will never be personally identifiable.

When you have finished you will be given a debriefing sheet which explains the purpose of the study and contains contact information for any follow-up questions you may have.

It is up to you to decide whether or not to take part. If you do decide to take part you will be given this information sheet to keep and be asked to sign a consent form.

Consent Form

Please complete this form after you have read the Information Sheet and/or listened to an explanation about the research.

Title of Study: Learning dynamics in the design of virtual artifacts

• Thank you for considering taking part in this research. You must read the Information Sheet before you agree to take part.

• If you have any questions arising from the Information Sheet, please ask the researcher before you decide whether to join in. You will be given a copy of this Consent Form to keep and refer to at any time.

• I understand that if I decide at any other time during the research that I no longer wish to participate in this project, I can notify the researchers involved and be withdrawn from it immediately (up until the point of publication of the study).

• I consent to the processing of my personal information for the purposes of this research study. I understand that such information will be treated as strictly confidential and handled in accordance with the provisions of the Data Protection Act 1998. Note that your name and contact details will not be stored anywhere in our records.

Participant's Statement:

I, ____ (PRINT YOUR NAME) agree that the research project named above has been explained to me to my satisfaction and I agree to take part in the study. I have read both the notes written above and the Information Sheet about the project, and understand what the research study involves.

Signed: Date:

Investigator's Statement:

I ____ (PRINT YOUR NAME) confirm that I have carefully explained the nature, demands and any foreseeable risks (where applicable) of the proposed research to the volunteer

Signed: Date:

DEBRIEFING SHEET

INVESTIGATOR'S NAME: **DR CLAUDIO TENNIE**
SCHOOL OF PSYCHOLOGY
UNIVERSITY OF BIRMINGHAM

STUDY TITLE: STUDYING CULTURAL TRANSMISSION IN VIRTUAL COMPUTER TASKS

Thank you for participating in this study. In this experiment you engaged in a computer-based learning task in which you designed your own 'virtual arrowheads'. The objective was to get your designs as close to a hidden optimal design as possible. Throughout the experiment you could use direct feedback from the environment to improve your arrowhead. We call this 'individual learning'. Depending on which experimental condition you were in (social learning or individual learning alone), you could also choose to copy the arrowhead of other members of the group. We call this 'social learning'. Another factor that we varied across participants was whether the hidden optimal design of the arrowhead left many cues in the environment, or few: either many of the peak's neighboring arrowhead designs produced rather high outcomes ("broad peaks"), or else you had to be very close to the optimal design before you gained high outcomes ("narrow peaks").

The purpose of this study was to discover if people would use less social learning when the environment did provide a lot of useful feedback ("broad peak" condition) than when it did not ("narrow peak" condition). The individual learning conditions (where no social learning options were given) were included as baseline conditions – i.e. to compare the social learning conditions to. Previous studies of this kind did not vary the types ofenvironment in this way – and the relatively low levels of social learning found in them might be explained by their usage of rather "broad peak" environments. If you are interested in this research, the references shown below contain more details.

QUESTIONS: If you have any further questions, or wish to be sent any future publications regarding this work, please contact Dr Claudio Tennie (Email: c.tennie@bham.ac.uk).

REFERENCES

Mesoudi, A. (2008) An experimental simulation of the "copy-successful-individuals" cultural learning strategy: Adaptive landscapes, producer-scrounger dynamics and informational access costs. *Evolution and Human Behavior, 29(5)*, 350-363. http://amesoudi.googlepages.com/Mesoudi_EHB_2008.pdf

McElreath, R., Bell, A. V., Efferson, C., Lubell, M., Richerson, P. J., & Waring, T. (2008). Beyond existence and aiming outside the laboratory: Estimating frequency-dependent and payoff-biased social learning strategies. *Philosphical Transactions of the Royal Society B*. http://xcelab.net/rmpubs/mcelreath et al phil trans B 2008 in press.pdf
